# Multi-Block Copolymer Membranes Consisting of Sulfonated Poly(*p*-phenylene) and Naphthalene Containing Poly(arylene Ether Ketone) for Proton Exchange Membrane Water Electrolysis

**DOI:** 10.3390/polym15071748

**Published:** 2023-03-31

**Authors:** Eui Jin Ko, Eunju Lee, Jang Yong Lee, Duk Man Yu, Sang Jun Yoon, Keun-Hwan Oh, Young Taik Hong, Soonyong So

**Affiliations:** 1 Energy Materials Research Center, Korea Research Institute of Chemical Technology (KRICT), Daejeon 34114, Republic of Korealjylee@krict.re.kr (J.Y.L.); dmyu@krict.re.kr (D.M.Y.); sjyoon@krict.re.kr (S.J.Y.); 2 Department of Polymer Engineering, Chungnam National University, Daejeon 34134, Republic of Korea

**Keywords:** multi-block copolymer, naphthalene-based oligomer, polymer electrolyte membrane water electrolysis

## Abstract

Glassy hydrocarbon-based membranes are being researched as a replacement for perfluorosulfonic acid (PFSA) membranes in proton exchange membrane water electrolysis (PEMWE). Here, naphthalene containing Poly(arylene Ether Ketone) was introduced into the Poly(*p*-phenylene)-based multi-block copolymers through Ni(0)-catalyzed coupling reaction to enhance *π-π* interactions of the naphthalene units. It is discovered that there is an optimum input ratio of the hydrophilic monomer and NBP oligomer for the multi-block copolymers with high ion exchange capacity (IEC) and polymerization yield. With the optimum input ratio, the naphthalene containing copolymer exhibits good hydrogen gas barrier property, chemical stability, and mechanical toughness, even with its high IEC value over 2.4 meq g^−1^. The membrane shows 3.6 times higher proton selectivity to hydrogen gas than Nafion 212. The PEMWE single cells using the membrane performed better (5.5 A cm^−2^) than Nafion 212 (4.75 A cm^−2^) at 1.9 V and 80 °C. These findings suggest that naphthalene containing copolymer membranes are a promising replacement for PFSA membranes in PEMWE.

## 1. Introduction

Hydrogen has gained significant attention as an eco-friendly fuel due to its high energy storage capacity and abundance in water [1]. However, the conventional hydrogen production processes, such as coal gasification and natural gas reforming, emit greenhouse gases, such as CO_2_ and CO [2]. To address this issue, proton exchange membrane water electrolysis (PEMWE) technology, which only utilizes water and electricity, has emerged as a promising technology for “green hydrogen” production [3,4,5]. A key component of a PEMWE cell is the proton exchange membrane (PEM), which conducts protons from the anode to the cathode and prevents the permeation of produced hydrogen to the anode side. The current state-of-the-art membrane material is PFSA, such as Dupont’s Nafion, due to its excellent proton conductivity and chemical stability for PEMWE applications. However, there are concerns about the use of PFSA membranes in PEMWE operations at temperatures above 90 °C in fully hydrated conditions, as their mechanical properties weaken due to their low *α*-transition temperature [6,7,8,9]. Additionally, due to their high hydrogen permeability, there are limitations in improving cell efficiency and there is a concern about explosion conditions on the oxygen-rich side [10,11,12,13,14]. Therefore, there is a need to develop alternative materials that exhibit good mechanical and chemical stabilities, and gas barrier properties, to overcome the limitations of PFSA membranes in PEMWE technology.

In the world of electrochemical applications, PFSA membranes are commonly used but are expensive. Researchers have been exploring cheaper and more adaptable alternatives, such as hydrocarbon-based membranes. These alternatives, such as sulfonated copolymers of polyarylene [15,16], polyphenylene [17,18], and polyimide [19], offer advantages, such as being cost-effective, and having controllable chemical and physical properties. These hydrocarbon-based ionomers have become popular for PEMWE operations due to their low hydrogen permeability and excellent thermal and mechanical stability. The development of hydrocarbon-based ionomers provides a cost-effective and versatile alternative to PFSA membranes in a range of electrochemical applications, including fuel cells and electrolysis cells [20]. Recently, Klose et al. reported that the sulfonated poly(phenylene sulfone) (SPPS) has a potential to replace PFSA in PEMWE system. SPPS exhibited low gas crossover and high proton conductivity, and the PEMWE performance was higher (3.5 A cm^−2^ at 1.8 V) than that of Nafion 115 (1.5 A cm^−2^ at 1.8 V) [20]. The sulfonated poly(ether-ketone) with hexafluorobisphenol A and difluoroketone monomer exhibited excellent mechanical strength, low swelling property, and high glass transition temperature (>150 °C), and showed the current density of 1.0 A cm^−2^ at 1.67 V and 80 °C [21]. The sulfonated polystyrene grafted ethylenetetrafluoroethylene (ETFE) membranes also showed better mechanical properties and low hydrogen crossover rates than those of Nafion [22]. Sulfonated Poly(arylene Ether Sulfone) (BPSH), which is a typical hydrocarbon-based ionomer, exhibited excellent physical and electrochemical characteristics, and the cell performance in PEMWE application with the current density of 1.1 A cm^−2^ at 1.6 V and 90 °C [23]. In the study of Han et al., the statistical BPSHs are tentatively more promising than the blocky BPSHs for PEMWE in the viewpoint of the ratio of proton conductivity to hydrogen gas permeability, even though the statistical ones showed less developed hydrophilic water channels [24]. In the recent study, our group applied the multi-block copolymers consisting of rigid hydrophilic sulfonated Poly(*p*-phenylene) (SPP) segments and flexible hydrophobic Poly(arylene Ether Ketone) oligomers for PEMWE. In the ionomer backbone, there is no hetero-atom linkage in the hydrophilic parts to enhance the chemical stability from the hydroxyl radical attack [25]. However, there are not many reports of hydrocarbon-based membranes, especially about the hydrophobic block modification for PEMWE application.

Here, the introduction of a naphthalene unit based on a fused pair of benzene rings into hydrophobic Poly(arylene Ether Ketone) oligomers has been explored to enhance *π-π* interactions. This modification results in improved hydrophilic-hydrophobic phase separation and physical properties similar to the previous studies [26,27,28,29,30,31]. The multi-block copolymers in this study were prepared through the Ni(0)-catalyzed coupling reaction of 3-sulfonated-2,5-dichloro benzophenone (SDCBP) and Cl-terminated naphthalene-based Poly(arylene Ether Ketone) (Cl-NBP, 6.4 kDa). The input weight ratio of SDCBP to Cl-NBP was controlled from 1:1 to 7:1, corresponding the target ion exchange capacity (IEC) from 1.5 to 2.7 meq g^−1^. The highest IEC (2.49 meq g^−1^) was achieved at the ratio of 1:5, where the multi-block copolymer showed higher proton conductivity and PEMWE cell performance (5.5 A cm^−2^) than Nafion 212 (4.75 A cm^−2^) at 1.9 V and 80 °C.

## 2. Materials and Methods

### 2.1. Materials

1,5-Dihydroxynaphthalene (1,5-DHN, 97%), potassium carbonate (K_2_CO_3_, ACS reagent, ≥9.0%) nickel(II) bromide (NiBr_2_, anhydrous, powder, ≥99.9%), triphenylphosphine (TPP, reagentplus, 99%), zinc dust (Zn, >98%), *N*,*N*-dimethylacetamide (DMAc, anhydrous, 99.8%), 1-methyl-2-pyrrolidinone (NMP, anhydrous, 99.5%), and toluene (anhydrous, 99.8%) were purchased from Sigma-Aldrich (St. Louis, MO, USA). 4,4′-Difluorobenzophenone (DFBP, 99%) was obtained from TCI (Nihonbashi-honcho, Chuo-ku, Japan), and 4-chloro-4′-fluorobenzophenone (CFBP) and SDCBP were purchased from IS CHEM (Cheongju, Republic of Korea). K_2_CO_3_ was dried at 100 °C, and TPP was recrystallized using hexane prior to use. Zn dust was stirred in 1.0 M HCl solution in diethyl ether for 5 min, then washed with diethyl ether several times, and dried at 110 °C under vacuum using Abderhalden’s drying apparatus. All the other reagents were used as received.

### 2.2. Synthesis of Cl-Terminated NBP (Cl-NBP)

The hydrophobic oligomer, OH-terminated NBP, was synthesized with 1,5-DHN and DFBP by condensation reaction. Then, 1,5-DHN (15.72 g, 95.22 mmol), DFBP (18.25 g, 82.79 mmol), K_2_CO_3_ (15.79 g, 114.26 mmol), DMAc (100 mL) and toluene (25 mL) were added to a 250 mL 4-neck round bottom flask equipped with a Dean-Stark trap, a condenser, an overhead mechanical stirrer, and an argon gas inlet. The mixture was heated to 160 °C, stirred for 3 h, then the temperature gradually increased to 165 °C for 1 h to remove water azeotropically from the reaction mixture. The mixture was cooled to room temperature (RT) after stirring for further 3 h at 165 °C. The reaction mixture was coagulated in deionized water and washed several times with hot deionized water and MeOH. Finally, the obtained hydrophobic oligomer, OH-NBP, was vacuum-dried at 80 °C for 24 h. OH-NBP (25 g, 3.91 mmol), CFBP (4.58 g, 19.53 mmol), K_2_CO_3_ (1.08 g, 7.81 mmol), and NMP were added to a 250 mL 3-neck round bottom flask equipped with an overhead mechanical stirrer and an argon gas inlet. The mixture was stirred at 130 °C for 16 h, and at 150 °C for 2 h. After cooling to RT, the reaction mixture was poured into deionized water and washed with hot deionized water and MeOH. Finally, the obtained Cl-NBP was vacuum-dried at 80 °C for 24 h.

### 2.3. Polymerization of SDCBP and Cl-NBP, and Polymer Characterization

Sulfonated Poly(*p*-phenylene) multi-block copolymers were synthesized by Ni(0)-catalyzed coupling reaction. In a 250 mL 3-neck round bottom flask equipped with an overhead mechanical stirrer, NiBr_2_ (0.07 eq. to SDCBP and Cl-NBP), TPP (7.0 eq. to NiBr_2_), and Zn (60.0 eq. to NiBr_2_) were added and dissolved in NMP. The color of the catalyst mixture was changed from yellowish brown to reddish dark brown while stirring at 80 °C for 1 h. After the catalyst mixture was activated, a mixture of SDCBP and Cl-NBP in NMP was injected into the flask containing the catalyst mixture at 80 °C using a syringe. The input weight ratio of SDCBP to Cl-NBP varied from 1:1 to 7:1. The reaction was stirred for 20 h at a constant temperature. After cooling to RT, the reaction mixture was poured into ethanol containing 10% HCl (2 L). After stirring several hours, the precipitated product was filtered and washed with hot ethanol three times, then vacuum-dried at 80 °C for 24 h. Based on the input ratio of SDCBP to Cl-NBP (1:1, 3:1, 5:1, and 7:1), the multi-block copolymers were named as SPPNBP_1, 3, 5, and 7, respectively.

^1^H- and ^19^F-nuclear magnetic resonance (NMR) spectra of the synthesized polymers were measured using 500 MHz DRX300 spectrometer (Bruker, Billerica, MA, USA) with dimethylsulfoxide-*d*6 and chloroform-*d*. The inherent viscosities (*ƞ*_inh_) of polymer solutions in NMP were measured at a concentration of 0.05 g dL^−1^ containing 0.05 M lithium bromide (LiBr) at 25 °C using a Cannon-Fenske routine viscometer. The number (*M*_n_) and weight average molecular weights (*M*_w_) and dispersity (*Ð*) of SPPNBP polymers were obtained by a gel permeation chromatography (GPC, YL9100, Young In, Seoul, Republic of Korea) equipped with a Shodex KF-805L column, YL9120 UV/Vis detector, and YL9112 isocratic pump at 50 °C. DMAc with 0.05 M LiBr was used as the eluent with a flow rate of 1.0 mL min^−1^.

### 2.4. Membrane Preparation and Characterization

SPPNBP polymers were dissolved in NMP (20 wt.%) at 80 °C for 10 h. The polymer solution was filtered with 5 μm polytetrafluoroethylene (PTFE) filter prior to use. The SPPNBP solutions were cast onto a pre-cleaned glass plate and dried at 60 °C for 15 h. Then, the membranes were immersed into 1 M H_2_SO_4_ aqueous solution for 24 h, followed by washing in deionized water for 24 h.

The dimensional stability of the membranes in water was quantified with the water uptake and dimensional changes with the weight and dimensional changes upon immersing in liquid water compared to the values at dried state. All membranes were completely dried under vacuum at 80 °C for 24 h, and swollen in liquid water at least 3 h.

The IEC values of the SPPNBP membranes were measured using a Metrohm 794 Basic Titrino Compact Titrator (Metrohm, Herisau, Switzerland) via titration method. The proton form dried membranes were stirred in 1.0 M NaCl solution for 24 h to exchange H^+^ ions to Na^+^ ions, then titrated with 0.01 M NaOH solution to pH 7.0.

The proton conductivity (*σ*) of the membranes was measured by an AC impedance spectroscopy, Solatron SI 1280B, at RT and 80 °C in water along the in-plane direction in the frequency range from 0.1 to 20 kHz using a four-point probe conductivity cell. The membranes were placed in a humidity chamber, SH-241 (ESPEC, Kita-ku, Osaka, Japan), and held for 1 h at the experimental condition before the measurement. The proton conductivity of the membranes was calculated by Equation (1) below [24]:

*σ* = *L*/(*R* × *S*), (1)

where *L* is the distance between electrodes (cm), *R* is the impedance (Ω), and *S* is the proton conducting cross-sectional area of the membranes (cm^2^).

The hydrogen permeability (*P*) of membranes was evaluated through the direct gas chromatography method using a gas chromatography (YL6500 GC, Young In, Seoul, Republic of Korea) with a 3 ft column (MolSieve 13X, Agilent, Santa Clara, CA, USA). Fully humidified (100% RH) hydrogen and argon flowed to the cathode and anode side, respectively, and the permeated hydrogen concentration to the argon side was measured by the GC. The *P* values were calculated with the following equation [32]:

*P* = (*C*_H_ × *v* × *t*)/(*A* × Δ*p*) (2)

where *C*_H_ is the permeated hydrogen concentration in the argon, *v* is the argon gas flow rate, *t* is the membrane thickness at 80 °C under 100% RH condition, *A* is the exposed area to the humid gases, and Δ*p* is the partial pressure difference of the hydrogen across the membranes.

The mechanical properties of the membranes were assessed by analyzing their stress–strain curves under both dry and wet conditions using a universal tensile machine (UTM) at RT. The evaluation was carried out following the ASTM-D638 standard at a cross head speed of 1.0 mm min^−1^.

The morphological characteristics of the membranes were analyzed using a transmission electron microscope (TECNAI G2 T-20S, FEI, Hillsboro, OR, USA). To prepare the samples for imaging, all the membranes were immersed in a 1.0 M lead acetate solution for 24 h to enable staining with lead ions. The stained samples were washed and carefully soaked in deionized water, then dried in a vacuum oven at 80 °C for 24 h. After drying, the samples were embedded in epoxy resin, sectioned into a thickness of 20 nm using a LEICA EM UC6 Ultra microtome, and placed on copper grids for imaging.

### 2.5. Preparation of Membrane Electrode Assembly (MEA) and PEMWE Single Cell Performance

The details of the MEA preparation are described in the previous our publication [24]. In brief, a gas diffusion electrode (5 cm^2^ GDE, CNL Energy) with Pt/C catalyst layer (CL) (Pt/C ratio: 0.4, 0.5 mg-Pt/cm^2^) and IrO_2_ CL (Nafion/IrO_2_ ratio: 0.08, 2.0 mg-IrO_2_/cm^2^) were decal-transferred to a membrane by a hot press machine (QM900S, QMESYS). For the anodic transport layer, a 5 cm^2^ Pt-coated Ti porous transport layer (PTL) was used.

With a MEA and a PTL, a PEMWE single cell was assembled, and its performance was recorded at 80 °C using a PEMWE station (CNL Energy). The voltage increased from 1.4 to 2.0 V with the scanning rate of 0.025 V min^−1^.

## 3. Results and Discussion

**Figure 1:**
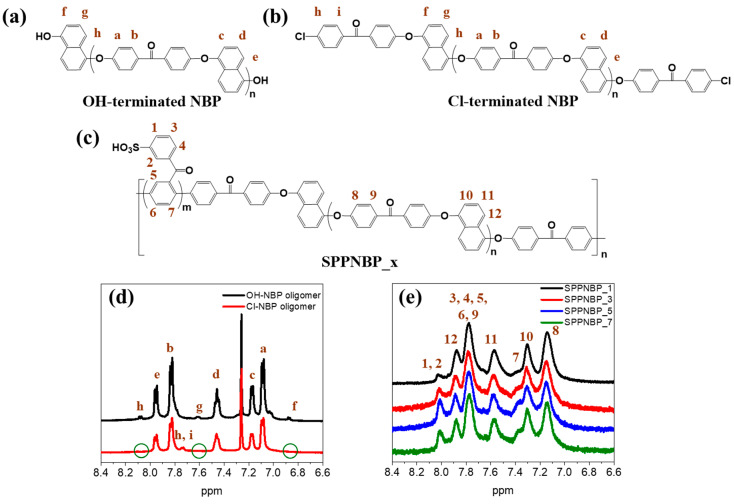
Chemical structures (**a**) OH-terminated NBP, (**b**) Cl-terminated NBP and (**c**) SPPNBP series), ^1^H-NMR spectra of (**d**) oligomers and (**e**) SPPNBP_1, 3, 5, and 7.

The hydrophobic oligomer, OH-NBP (Figure 1a), was synthesized by nucleophilic aromatic substitution reaction as shown in Scheme S1. Based on our previous research, the hydrophobic block length effect was optimized and, therefore, we aimed to achieve a molecular weight of 6.5 kDa for the oligomer [25,33]. Through the end-group analysis using the ^1^H-NMR spectrum (Figure 1d), the molecular weight of OH-NBP (*M*_NBP_) was evaluated. The peak integrations of *g* and *a* proton peaks of OH-NBP were evaluated to have the degree of polymerization, *n*. The *n* value was found to be 18.4 corresponding *M*_NBP_ = 6.4 kDa, which is close to the targeted molecular weight. As shown in Figure 1d, with the reaction of OH-NBP and CFBP, *f*, *g,* and *h* proton peaks of OH-NBP at 8.08 ppm, 7.61 ppm, and 6.87 ppm, respectively, disappeared, but *h* and *i* proton peaks of Cl-NBP (Figure 1b) appeared at 7.73−7.75 ppm. As depicted in Scheme S1, the four different SPPNBP multi-block copolymers were synthesized by Ni(0)-catalyzed cross-coupling reaction. In all input ratios, copolymers were well synthesized as indicated by the viscosity of the polymer solutions, and the molecular weight of the polymers measured by a GPC. The inherent viscosity (*η*_inh_) values for SPPNBP_1, 3, 5, and 7 were 0.60 dL g^−1^, 0.88 dL g^−1^, 1.12 dL g^−1^, and 0.84 dL g^−1^, respectively, and the molecular weights (*M*_n_) of 33.5 kDa, 45.2 kDa, 59.6 kDa, and 49.2 kDa, respectively. Figure 1e displays the ^1^H-NMR spectra of the produced polymers. The spectra indicate that all the polymers show the characteristic proton peaks of both SDCBP (1−7) and Cl-NBP (8−12). The molecular weight information is listed in Table 1.

Interestingly, however, as shown in Figure 1e, the intensity of peaks from SDCBP (1−7) compared to that of the hydrophobic blocks increases with the increasing input fraction of SDCBP up to 1:5, but decreases as the input fraction is further increased to 1:7. The IEC values from the titration method also showed the monotonic increase up to 1:5 (SPPNBP_5) as 2.49 meq g^−1^ from the value of SPPNBP_1 (1.10 meq g^−1^), but the value decreased to 2.06 meq g^−1^ for SPPBNP_7, which is similar to the value of SPPNBP_3 (2.05 meq g^−1^). This trend might be attributed to the loss of high IEC oligomers and copolymers during the ethanol precipitation stage [25,34]. Studies on copolymerization involving SDCBP and hydrophobic oligomers suggest that significant amounts of high IEC products are lost after the ethanol extraction, particularly when using a higher SDCBP/hydrophobic oligomer input ratio. During the copolymerization between hydrophobic Cl-NBP oligomers and hydrophilic SDCBP monomers, the reactivity of -Cl groups in the oligomers is lower than that of the monomers due to their low mobility [35]. Therefore, after the hydrophilic blocks are formed from SDCBP monomers, they are able to be involved in coupling reactions with hydrophobic Cl-NBP oligomers. However, without the sufficient copolymerization of the hydrophilic blocks with hydrophobic oligomers, homo-polymerized hydrophilic blocks and high IEC SPPNBP copolymers might be washed away by the ethanol washing process [25]. At high monomer/oligomer input ratio, it is likely that some of the resulting products would be high IEC copolymers or hydrophilic blocks, which may be challenging to precipitate using ethanol. In this study as well, it was observed that as the input ratio increased, the polymerization yields after the ethanol extraction decreased. Specifically, the yields of SPPNBP_1, 3, 5, and 7 were 69.1%, 57.1%, 51.1%, and 12.9%, respectively.

**Figure 2:**
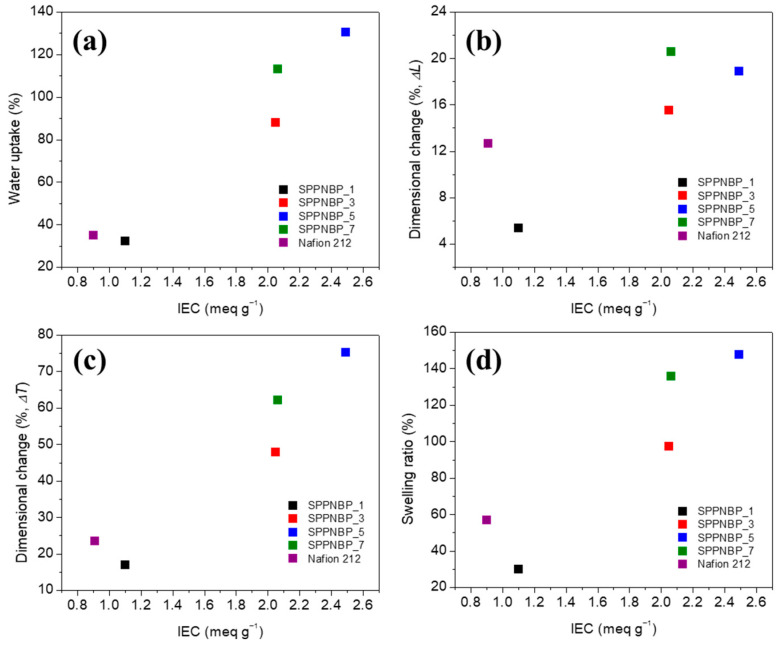
(**a**) Water uptake, dimensional changes of (**b**) in-plane (Δ*L*), and (**c**) through-plane (Δ*T*) directions, and (**d**) swelling ratio of SPPNBP membranes at 80 °C.

There is a strong correlation between the water uptake (WU) of a membrane and its IEC, such that membranes with higher IEC tend to exhibit higher WU. Figure 2 shows the WU of the SPPNBP membranes, and the dimensional changes upon swelling at 80 °C, the PEMWE operation temperature. The WU values of the SPPNBP membranes increase from 32.3% (SPPNBP_1) to 130.4% (SPPNBP_5) with the IEC values, as shown in Figure 2a. The dimensional changes of the SPPNBP membranes exhibited a similar trend to the WU values. As the IEC values increase, both the through-plane direction (Δ*T*) (Figure 2b), in-plane direction changes (Δ*L*) (Figure 2c), and volumetric changes (Figure 2d) increase in the SPPNBP membranes. All membranes exhibited greater swelling along the through-plane direction compared to the in-plane direction, which is a characteristic feature of ion conducting block copolymer membranes [24,36]. Although SPPNBP_3 and SPPNBP_7 exhibit comparable IEC values, there are apparent distinctions in their WU and dimensional changes, which could be attributed to variations in the length of their hydrophilic blocks. As stated earlier, when the monomer/oligomer ratio is higher, longer hydrophilic blocks are anticipated to be created during the coupling reaction with the oligomers. Copolymer membranes with longer hydrophilic blocks are known to absorb and bind to water more readily because they have higher accessibility to the hydrophilic domains. Based on the assumptions that the hydrophilic blocks (Block A) have grown sufficiently to participate in coupling reactions with the hydrophobic block (Block B), and that the coupling occurs alternately between the hydrophilic and hydrophobic blocks (−A-B-A-B-A-B-A−) while completing the coupling reaction with the hydrophilic block ends (A−(B-A)_n_−B-A), a rough estimate suggests that the minimum length of the hydrophilic block (i.e., the average molecular weight of the hydrophilic blocks, *M*_n,SPP_) required to achieve the given *M*_n_ and IEC of the copolymers is longer for SPPNBP_7 (*M*_n,SPP_ = 9.8 kDa) than for SPPNBP_3 (*M*_n,SPP_ = 9.4 kDa). 

To observe the effect of the naphthalene unit on membrane swelling, the swelling behavior of SPPNBP_3 was compared to that of the SPP-*b*-poly(ether sulfone ether ketone) multi-block copolymer (SPP-PSK), which has a biphenyl sulfone unit instead of a naphthalene unit [33]. At room temperature, the SPP-PSK membrane showed WU = 68 wt.%, Δ*T* = 44%, and Δ*L* = 13%, which are higher than the values for SPPNBP_3 (WU = 56 wt.%, Δ*T* = 33%, and Δ*L* = 7%), even though SPPNBP_3 has higher IEC value (2.05 meq g^−1^) than SPP-PSK (1.8 meq g^−1^). From this comparison, the reduced dimensional changes in water with the naphthalene unit might be attributed to the enhanced *π-π* interactions.

**Figure 3:**
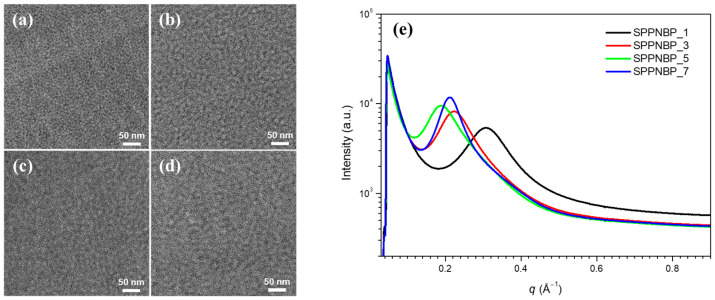
TEM images of (**a**–**d**) SPPNBP membranes, and (**e**) SAXS intensity profiles.

To observe the cross-sectional morphology of the SPPNBP membranes, they were soaked in a solution of 1.0 M lead acetate to stain the hydrophilic domains. Transmission electron microscopy (TEM, Tecnai G2 T-20s) was used to take the TEM images of the samples, as seen in Figure 3a–d. The images show the hydrophilic domains, which appear dark due to the lead staining, while the hydrophobic domains appear bright [24,25]. The observed results indicate that all the samples exhibit well-defined and continuous hydrophilic channels. Furthermore, the measured width (*w*) of the channels increases in the order of the input ratio of the monomer/oligomer. The widths of the channels for SPPNBP_1, 3, and 5 are measured to be *w* = 1.6 ± 0.3 nm, 9.0 ± 0.5 nm, and 11.6 ± 0.5 nm, respectively. However, in the case of SPPNBP_7, the trend was not consistent as its width was reduced to *w* = 10.5 ± 2.1 nm, which is narrower than that of SPPNBP_5, but wider than that of SPPNBP_3. This width of hydrophilic channels in the SPPNBP series may be possibly explained by the variation in hydrophilic block length in the multi-block copolymers. As mentioned earlier, longer hydrophilic blocks may be formed at higher monomer/oligomer ratios. However, when the monomer and oligomer ratio is excessively high, only copolymers with relatively lower IEC values and longer hydrophilic blocks in the mixture may be fractionated. The TEM results provide evidence that there is indeed a variation in the hydrophilic block length during the coupling reactions and precipitation steps.

Figure 3e displays the SAXS spectra of the SPPNBP series, which were obtained at the PLS-II 3C beamline of PAL. The hydrophilic domain spacing (*d* = 2*π*/q*, where q* is the scattering vector at the maximum scattering intensity) is on the same order as the variation in the hydrophilic block length. The values of *d* for SPPNBP_1, 3, 5, and 7 are 2.0 nm, 2.8 nm, 3.4 nm, and 3.0 nm, respectively.

The TEM images of SPPNBP_3 and SPP-PSK (shown in Figure S1a,b) reveal that the phase separation between the hydrophobic and hydrophilic domains is more evident in the SPPNBP copolymers than in SPP-PSK, possibly due to the improved aggregation of naphthalene units in the hydrophobic domains. Additionally, the incorporation of naphthalene units has led to a clearer evolution of the hydrophilic channel, as confirmed by the sharper scattering peak observed in the SAXS spectra of SPPNBP_3 compared to SPP-PSK (shown in Figure S1c).

**Figure 4:**
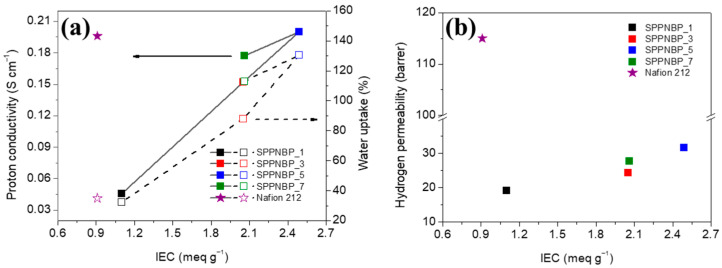
(**a**) The relation between the proton conductivities and the water uptake values of SPPNBP membranes and Nafion 212 membranes depending on IEC at 80 °C. (**b**) Hydrogen permeability of the membranes at 80 °C.

Proton conductivity (*σ*) of a PEM is one of the key parameters determining PEMWE performance and is mainly affected by IEC and membrane morphology [37]. The proton conductivities of SPPNBP membranes were measured at 80 °C. As expected, the *σ* values increased with IEC and WU (Figure 4a). SPPNBP_1, which has the lowest IEC, 1.10 meq g^−1^, shows *σ* = 0.046 S cm^−1^, but the conductivity increases to *σ* = 0.152 (SPPNBP_3) and 0.200 S cm^−1^ (SPPNBP_5). For SPPNBP_5, the proton conductivity was comparable to Nafion 212 and was the highest in the SPPNBP series. With the NBP blocks, high IEC value is necessary to have the comparable *σ* value of Nafion 212 (*σ* = 0.196 S cm^−1^), compared to other SPP based multi-block copolymers. The inclusion of rigid and planar naphthalene units in the copolymers is advantageous as it helps to impede changes in the dimensions of liquid water [33]. However, the incorporation of hydrophobic blocks based on naphthalene results in a slight reduction in proton conductivity, as it decreases the amount of water uptake. When comparing SPPNBP_7 to SPPNBP_3, it is likely that the wider hydrophilic domains in SPPNBP_7 contributed to the higher *σ* value of 0.177 S cm^−1^. 

In PEMWE, the primary product, hydrogen, tends to cross over to the oxygen-rich side mainly through the hydrophilic channels that contain liquid water where the protons are transported. Unlike protons, the high hydrogen crossover is problematic in PEMWE operation due to the undesirable mixing of hydrogen and oxygen gases, which causes an explosion, and low cell efficiency and low hydrogen production yield. Therefore, low gas permeability is one of the key factors to enhance the performance of PEMWE. 

The hydrogen permeability (*P*) of the SPPNBP membranes was measured at 80 °C and 100% RH using a GC. As shown in Figure 4b, the *P* values of all membranes are considerably lower below 32 barrer compared to Nafion 212, which had a *P* value of 115.0 barrer. It should be noted here that similar to the proton conduction, SPPNBP_5 (IEC = 2.49 meq g^−1^) showed the highest *P* value as 31.7 barrer, which is even lower than the BPSH membrane with the IEC value of 2.27 meq g^−1^ [24]. This remarkable hydrogen barrier property of the SPPNBP membranes is probably due to the enhanced dimensional stability in liquid water and the reduced free volume of the hydrophobic domains by peculiar structural stacking of naphthalene units.

With the values of proton conductivity and hydrogen permeability, the proton selectivity to hydrogen gas (*ξ*) was evaluated as an indicator for the proper PEM for PEMWE operation in the viewpoint of performance and safety. The *ξ* values of SPPNBP_1, 3, 5, and 7 are 2.4 mS cm^−1^ barrer^−1^, 6.2 mS cm^−1^ barrer^−1^, 6.3 mS cm^−1^ barrer^−1^, and 6.4 mS cm^−1^ barrer^−1^, respectively. Compared to Nafion 212 (*ξ* = 1.7 mS cm^−1^ barrer^−1^), all SPPNBP series showed higher selectivity values, and especially, the *ξ* values of SPPNBP_3, 5, and 7 was roughly 3.6 times higher than Nafion 212.

Despite hydrocarbon-based membranes demonstrating superior proton selectivity when compared to PFSA membranes, the adoption of hydrocarbon-based membranes as an alternative to PFSA membranes is hindered by their poor chemical stability, particularly in regard to oxidative stability [38]. To address the chemical stability of the SPPNBP samples, the oxidative stability test was conducted with Fenton reagent [24]. After immersing the membranes in Fenton’s reagent (3 wt% H_2_O_2_ and 2 ppm FeSO_4_) at 60 °C for 6 h, the residual weight was measured. Metal cations like Fe^2+^ and Fe^3+^ can break down hydrogen peroxide into hydroxyl (•OH) and hydroperoxyl (●OOH) radicals through the Fenton reaction. These radicals can speed up the degradation of both the sulfonic acid groups and the main backbone of membranes, leading to a decrease in their molecular weights and IEC values [38,39,40,41]. The residual weights after the test were 98.7%, 92.2%, 89.1%, and 95.5% for SPPNBP_1, 3, 5, and 7, respectively, which is proportional to the IEC values. Despite having high IEC values, the SPPNBP series exhibited relatively good chemical stability, particularly for SPPNBP_3, 5, and 7, even though their residual weights were lower than that of Nafion 212 (which had a residual weight of 99.4%). In contrast, when the same experiment was performed with SPP-PSK (which had an IEC of 1.79 meq g^−1^), only 77.0% of the membrane remained. These results suggest that the naphthalene units in the SPPNBP series may have contributed to enhancing the chemical stability of the membranes. 

**Figure 5:**
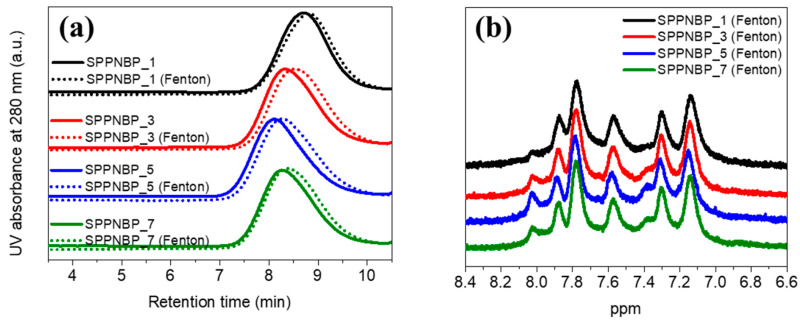
(**a**) GPC profiles of SPPNBP series before (solid lines) and after Fenton test (dotted lines). (**b**) ^1^H-NMR spectra of SPPNBP membranes after the Fenton test.

In addition to measuring the residual weight, the remaining membranes after the Fenton test were also analyzed using GPC traces and ^1^H-NMR spectra. The results of these analyses are presented and summarized in Figure 5 and Table 2. The GPC curves of all samples after the test (dotted line) were shifted to the lower molecular weight region compared to those before the test (solid line), as shown in Figure 5a. After the test, the *M*_n_ values decreased by 14% to 22%, and the *Ð* values increased by 3% to 6% for the membranes. The results obtained can be attributed to main-chain scission that occurred in all SPPNBP membranes. As observed in the ^1^H-NMR spectra after the Fenton test (Figure 5b), the peaks for the hydrophilic SPP blocks (7.4 ppm to 8.0 ppm) were slightly reduced compared to Figure 1e. To compare the changes in IEC values, the values were calculated from the integral ratio of both ^1^H-NMR spectra (Figure 1e and Figure 5b) and listed in Table S1. The IEC values from ^1^H-NMR showed quite different values when comparing the values by titration method as listed in Table S1 due to the slight overlaps between adjacent peaks. However, the tendency was the same for each polymer (SPPNBP_5 > 7 ≈ 3 > 1). The IEC values decreased by 15.8%, 15.6%, and 18.0% for SPPNBP_3, 5, and 7, respectively, while SPPNBP_1 showed an increased IEC value by 27.1%. It is known that poly(arylene ether) is relatively weak to chemical degradation, whereas poly(phenylene) based structures show higher stability [42]. Therefore, SPPNBP_1, consisting of relatively lower sulfonated SPP block content than the other SPPNBP membranes, is more likely to be attacked on NBP blocks containing ether (−O−) linkages rather than the SPP blocks, and showed higher IEC value after the Fenton test. 

**Figure 6:**
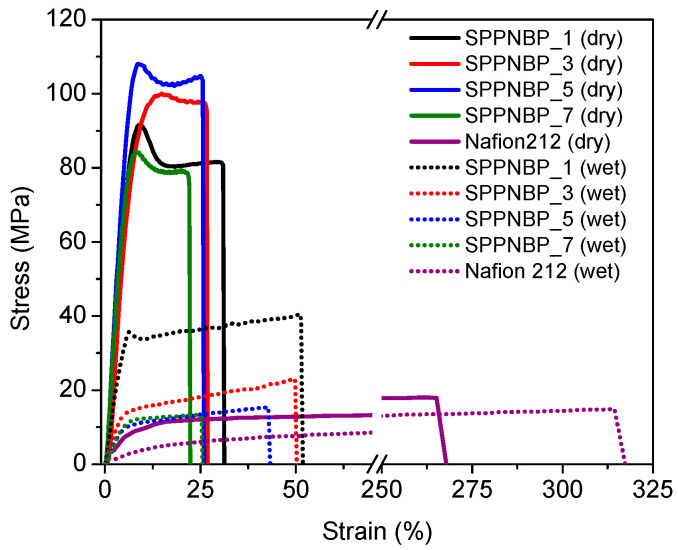
Strain-stress curves of SPPNBP membranes and Nafion 212 membranes.

The SPPNBP membranes were subjected to a tensile test by a UTM, and the results were compared to Nafion 212 membrane. As shown in Figure 6 and Table 2, all SPPNBP membranes exhibited outstanding maximum tensile strength (ranging from 84.3 MPa to 108.0 MPa) and Young’s modulus (ranging from 1.48 GPa to 1.86 GPa) compared to Nafion 212 membrane (with a tensile strength of 18.0 MPa and Young’s modulus of 130 MPa). The mechanical properties of the membranes were greatly influenced by properties, such as molecular weight, polymer structure, and the percentage of sulfonic acid groups in the main chain. The introduction of rigid naphthalene units into the polymer backbone improved the tensile strength of the SPPNBP membranes compared to other types of multi-block copolymers without naphthalene units [33,34]. Despite having the highest IEC value (2.49 meq g^−1^) among the SPPNBP series, SPPNBP_5 performed exceptionally well, showing tensile strength of 108.0 MPa and Young’s modulus of 1.86 GPa. This was due to a well-balanced ratio between hydrophilic and hydrophobic blocks, and a relatively high molecular weight. The elongation at break for this series was between 22.4% to 31.3%. Typically, polymers with only a Poly(*p*-phenylene) based main-chain are rigid and brittle, resulting in high tensile stress but limited elongation, usually below 20%. The improved elongation at break values for the SPPNBP series suggest that these polymers have enhanced toughness and flexibility compared to pure Poly(*p*-phenylene) based materials [43,44,45,46]. Poly(arylene Ether Sulfone) and Poly(arylene Ether Ketone)-based ionomers are generally known to have flexible structures due to the presence of ether (−O−) linkages. These results are good elongation, but low tensile strength [35,46,47]. However, the combination of the naphthalene-based hydrophobic Poly(arylene Ether Ketone) block with the sulfonated Poly(*p*-phenylene) block had a complementary linkage that could overcome the brittleness of the polymer main chain, resulting in improved tensile strength and elongation despite its rigid structure. The results showed that as the ratio of the naphthalene-based NBP block increased (SPPNBP_1 > 3 > 5), the tensile strength and elongation values increased [29,30,31,37,38]. Surprisingly, SPPNBP_7 showed the lowest tensile strength and elongation in the series, despite having a higher molecular weight than SPPNBP_3, which had similar IEC values, but a larger ionic domain size. As discussed earlier, the poor mechanical properties of SPPNBP_7 may have been due to a high content of SPP blocks in the main chain, resulting in relatively longer segments of rigid and brittle SPP blocks. As indicated by the UTM results, their membrane condition was different, although SPPNBP_3 and SPPNBP_7 had similar IEC values.

During operation in PEMWE conditions, the membranes are fully hydrated, and, therefore, do not experience hydration or dehydration stress like in PEMFCs. Therefore, to ensure long-term stability and durability, it is important to consider the mechanical properties, particularly the maximum tensile strength, of the fully hydrated membranes [37,48,49,50]. 

When exposed to water, the tensile stress values of SPPNBP membranes decreased, while the elongation at break increased. Specifically, the values of SPPNBP_1, 3, 5, and 7 were 40.4 MPa, 22.9 MPa, 15.3 MPa, and 13.2 MPa, respectively, in the presence of water. Despite this decrease, the SPPNBP membranes still had higher tensile strength than Nafion 212, except for SPPNBP_7, with up to 2.7 times higher tensile strength. The elongation at break values for the SPPNBP membranes (SPPNBP_1, 3, 5, and 7) in the presence of water were 51.9%, 50.3%, 43.3%, and 25.3%, respectively.

**Figure 7:**
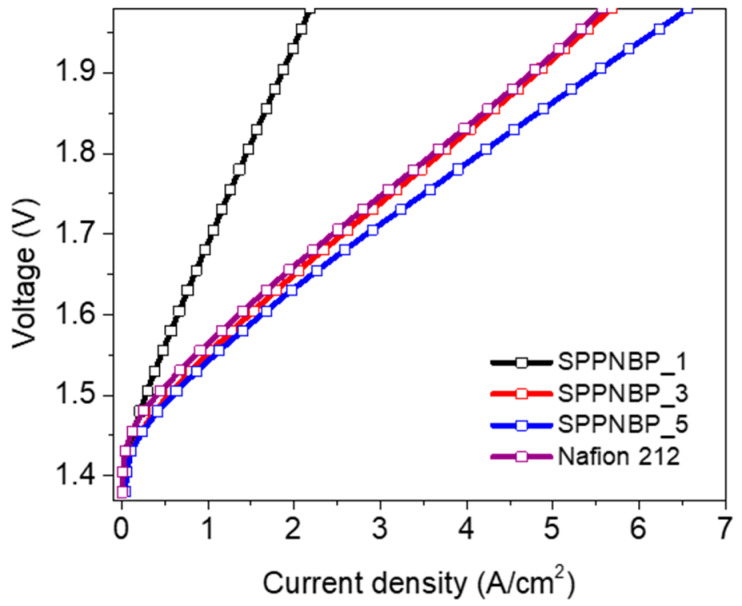
Polarization curves of SPPNBP_1, 3, and 5 membranes, and Nafion 212 membrane-based PEMWE single cells at 80 °C.

The PEMWE single cell performance was evaluated at 80 °C under the ambient pressure with SPPNBP_1, 3, and 5 membranes, and Nafion 212 membrane. In the case of SPPNBP_7, the cell performance was not measured because it had relatively low mechanical properties to introduce to a PEMWE single cell, which was easy to tear and fragile. The thickness of the SPPNBP membranes was in the range of 42 μm to 57 μm, which is similar to the thickness of Nafion 212 (50 μm). Figure 7 shows the polarization curves of the PEMWE single cells. At the same voltage, the current density is higher for the membrane having higher IEC value. For example, at 1.9 V, SPPNBP_5 exhibits 5.5 A cm^−2^, but SPPNBP_3 and 1 show lower current densities of 4.8 cm^−2^ and 1.9 A cm^−2^, respectively. With SPPNBP_5, especially the single cell performed better than Nafion 212 (4.8 A cm^−2^ at 1.9 V). 

## 4. Conclusions

This research involved synthesizing various types of SPPNBP membranes, which are made up of both hydrophilic SPP and hydrophobic NBP blocks, with different ratios of hydrophilic monomer to hydrophobic oligomer. All of the polymers were successfully synthesized with an ion exchange capacity (IEC) ranging from 1.10 meq g^−1^ to 2.49 meq g^−1^, using a Ni(0)-catalyzed coupling reaction. It was discovered that there is a maximum IEC value that can be achieved through the coupling reaction of the monomer and oligomer due to their different reactivity and precipitation step. To achieve high IEC values to compete with commercially available PFSA membranes, the input ratios of 1:3 and 1:5 were chosen as the optimal sets with a polymerization yield of over 50%. By introducing naphthalene units into the hydrophobic oligomers, the dimensional changes in liquid water, hydrogen gas barrier property, and mechanical toughness were improved, even with their high IEC values. Specifically, SPPNBP_5 membrane had higher proton conductivity at 0.2 S cm^−1^, but lower hydrogen permeability at 31.7 barrer than Nafion 212 at 80 °C. These improved properties resulted in 3.6 times higher proton selectivity to hydrogen gas than Nafion 212. The PEMWE single cells using SPPNBP_3 and 5 membranes performed well with current densities of 4.8 and 5.5 A cm^−2^ at 1.9 V and 80 °C, which was a higher performance than Nafion 212 (4.75 A cm^−2^) under the same conditions.

## Figures and Tables

**Table 1:** The properties of SPPNBP multi-block copolymers and Nafion 212.

	*η*^*a*^ (dL g^−1^)	Molecular Weight Information			IEC *^b^* (meq g^−1^)	Proton Conductivity (S cm^−1^)		Hydrogen Permeability *^c^* (S cm^−1^)
		*M*_n_ (kDa)	*M*_w_ (kDa)	*Ð*		RT	80 °C	
SPPNBP_1	0.60	33.5	51.3	1.5	1.10	1.09	0.97	0.046
SPPNBP_3	0.88	45.2	75.1	1.7	2.05	1.57	1.26	0.152
SPPNBP_5	1.12	59.6	105.1	1.8	2.49	1.71	1.22	0.200
SPPNBP_7	0.84	49.2	81.6	1.7	2.06	1.53	1.09	0.177
Nafion 212	−	−	−	−	0.91	1.55	1.09	0.196

*^a^* Measured at a concentration of 0.05 g dL^−1^ containing 0.05 M lithium bromide (LiBr) at 25 °C; *^b^* IEC was measured by the titration method; *^c^* Measured at 80 °C and 100% RH.

**Table 2:** The Fenton test results, and the mechanical properties of SPPNBP membranes.

	Molecular Weight Information after Fenton Test			Residual Weight (%)	Young’s Modulus (GPa)		Tensile Strength (MPa)		Elongation at Break (%)	
	*M*_n_	*M*_w_	*Ð*		Dry	Wet	Dry	Wet	Dry	Wet
SPPNBP_1	28.9	46.6	1.6	98.7	1.48	0.71	91.6	40.4	31.3	51.9
SPPNBP_3	35.6	60.7	1.7	92.2	1.66	0.32	100.1	22.9	27.0	50.3
SPPNBP_5	46.7	84.7	1.8	89.1	1.86	0.23	108.0	15.3	25.9	43.3
SPPNBP_7	42.0	72.5	1.7	95.5	1.55	0.26	84.3	13.2	22.4	25.6
Nafion 212	−	−	−	99.4	0.13	0.04	18.0	14.8	267.9	317.3

## Data Availability

Not applicable.

## Supplementary Materials

The following supporting information can be downloaded at: https://www.mdpi.com/article/10.3390/polym15071748/s1, Scheme S1: Synthesis procedure of OH-terminated NBP (**A**), Cl-terminated NBP (**B**) and SPPNBP copolymers; Figure S1: TEM images of (**a**) SPPNBP_3 and (**b**) SPP-PSK membranes, and (**c**) SAXS intensity profiles of SPPNBP_3 and SPP-PSK copolymers; Table S1: The IEC values of SPPNBP membranes through the titration and 1H NMR methods.
