# Supplementary material for: Multi-Block Copolymer Membranes Consisting of Sulfonated Poly(p-phenylene) and Naphthalene Containing Poly(arylene Ether Ketone) for Proton Exchange Membrane Water Electrolysis
Source: Polymers (Basel). 2023 Mar 31;15(7):1748. doi: 10.3390/polym15071748 (PMC10097103; doi:10.3390/polym15071748)
Supplement: Supplementary file 1 [file polymers-15-01748-s001.zip › polymers-2304654-supplementary.pdf]

polymers

MDPI

Supplementary Information

Multi-block copolymer membranes consisting of sulfonated poly(*p*-phenylene) and naphthalene containing poly(arylene ether ketone) for proton exchange membrane water electrolysis

Eui Jin Ko [1,†], Eunju Lee [1,2,†], Jang Yong Lee [1], Duk Man Yu [1], Sang Jun Yoon [1], Keun-Hwan Oh [1,*], Young Taik Hong [1,*], and Soonyong So [1,*]

[1] Energy Materials Research Center, Korea Research Institute of Chemical Technology (KRICT), Daejeon 34114, South Korea; ljylee@krict.re.kr (J.Y.L.); dmyu@krict.re.kr (D.M.Y.); sjyoon@krict.re.kr (S.J.Y.); khoh@krict.re.kr (K.H.O.), ythong@krict.re.kr (Y. T. H.), syso@krict.re.kr (S.S.)

[2] Department of Polymer Engineering, Chungnam National University, Daejeon 34134, South Korea; hwanyeop@krict.re.kr (E.L.)

* Correspondence: khoh@krict.re.kr (K.H.O.); ythong@krict.re.kr (Y. T. H.); syso@krict.re.kr (S.S.)

† These authors contributed equally to this work

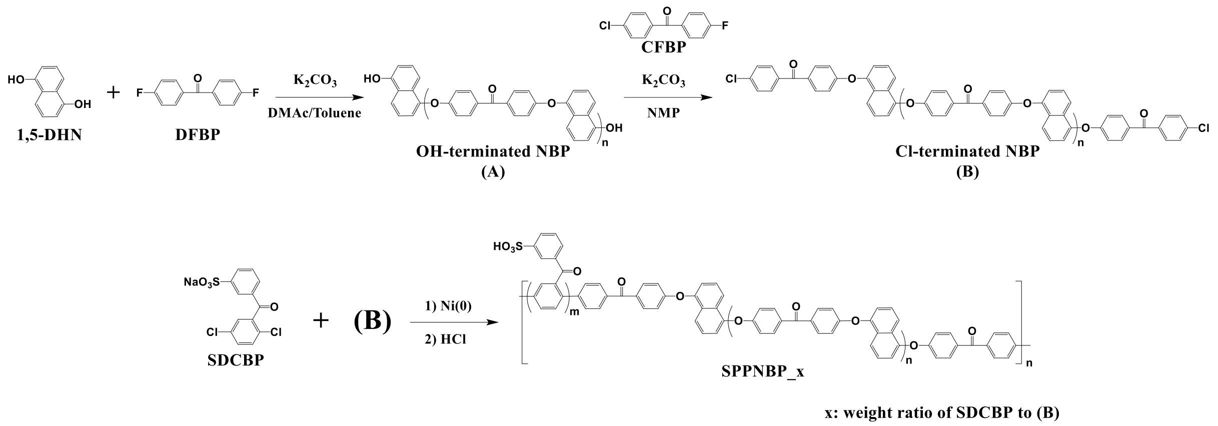

Scheme S1. Synthesis procedure of OH-terminated NBP (A), Cl-terminated NBP (B) and SPPNBP copolymers.

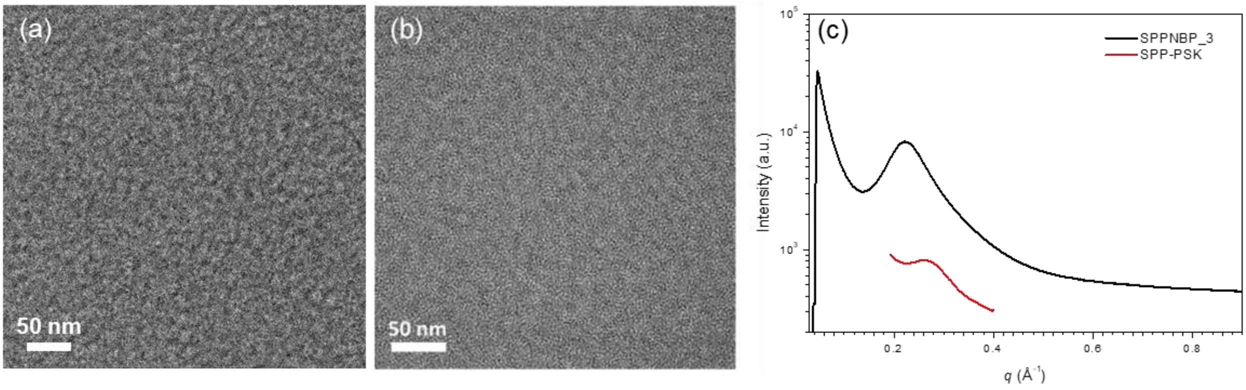

Figure S1. TEM images of (a) SPPNBP_3 and (b) SPP-PSK membranes, and (c) SAXS intensity profiles of SPPNBP_3 and SPP-PSK copolymers. (b) and (c) are reproduced from ref [1]. Copyright 2019 ACS.

Table S1. The IEC values of SPPNBP membranes through the titration and ^{1}H NMR methods.

	IEC (meq g^{-1})			Ratio [c] (%)
	Titration [a]	^{1}H NMR [a]	^{1}H NMR [b]	
SPPNBP_1	1.10	0.85	1.08	27.1
SPPNBP_3	2.05	1.46	1.23	−15.8
SPPNBP_5	2.49	1.73	1.46	−15.6
SPPNBP_7	2.06	1.50	1.23	−18.0

[a] The values before the Fenton's test.
[b] The values after the Fenton's test.
[c] Ratio (%) = $(IEC_{after} - IEC_{before})/IEC_{before} \times 100$ (%)

1. Hong, S. H.; Cha, M. S.; Hong, S.-K.; Oh, S.-G.; Lee, J. Y. Structural Effect of the Hydrophobic Block on the Chemical Stability of Ion-Conducting Multiblock Copolymers for Flow Battery. ACS Sustain. Chem. Eng. 2019, 7 (20), 17088–17099. https://doi.org/10.1021/acssuschemeng.9b03182.
